# Hygrothermal Properties and Performance of Bio-Based Insulation Materials Locally Sourced in Sweden

**DOI:** 10.3390/ma17092021

**Published:** 2024-04-26

**Authors:** Oskar Ranefjärd, Paulien B. Strandberg-de Bruijn, Lars Wadsö

**Affiliations:** Department of Building and Environmental Technology, Lund University, 221 00 Lund, Sweden

**Keywords:** bio-based, insulation, DVS, MBV, TPS, hot box, sorption calorimetry, wood fibre, eelgrass, grass

## Abstract

In recent years, there has been a paradigm shift in the building sector towards more sustainable, resource efficient, and renewable materials. Bio-based insulation derived from renewable resources, such as plant or animal fibres, is one promising group of such materials. Compared to mineral wool and polystyrene-based insulation materials, these bio-based insulation materials generally have a slightly higher thermal conductivity, and they are significantly more hygroscopic, two factors that need to be considered when using these bio-based insulation materials. This study assesses the hygrothermal properties of three bio-based insulation materials: eelgrass, grass, and wood fibre. All three have the potential to be locally sourced in Sweden. Mineral wool (stone wool) was used as a reference material. Hygrothermal material properties were measured with dynamic vapour sorption (DVS), transient plane source (TPS), and sorption calorimetry. Moisture buffering of the insulation materials was assessed, and their thermal insulation capacity was tested on a building component level in a hot box that exposed the materials to a steady-state climate, simulating in-use conditions in, e.g., an external wall. The tested bio-based insulation materials have significantly different sorption properties to stone wool and have higher thermal conductivity than what the manufacturers declared. The hot-box experiments showed that the insulating capacity of the bio-based insulators cannot be reliably calculated from the measured thermal conductivity alone. The results of this study could be used as input data for numerical simulations and analyses of the thermal and hygroscopic behaviour of these bio-based insulation materials.

## 1. Introduction

In recent years, there has been a paradigm shift in the building sector towards more sustainable, resource efficient, and renewable materials. The UN has identified local, low-embodied-carbon materials, such as bio-based building materials, as one of the focus topics for achieving climate neutrality in the building sector [1]. Bio-based insulation materials are derived from renewable resources such as plant or animal fibres. They offer several benefits over insulation materials from non-renewable and fossil-based resources. As they are derived from renewable resources, they can be replenished and are not subject to depletion like fossil fuel-based alternatives. Compared to conventional building materials, bio-based materials tend to have lower embodied greenhouse gas emissions [2]. This generally makes them a more sustainable choice for building materials and reduces the building sector’s environmental impact. Using locally sourced bio-based insulation materials can positively impact the local economy by creating jobs and supporting local businesses. This also reduces their environmental impact, as transportation distances are shorter [3]. Conventional insulation materials, such as mineral wool and polystyrene-based insulation materials, often have a significant environmental footprint, especially in the production stage [4,5,6]. 

In Sweden, new legislation implemented in January 2022 requires new buildings to declare their climate emissions when applying for a building permit [7]. Thus far, only a declaration of the climate emissions from a building’s production and construction stage (modules A1 to A5 according to standard [8]) is required [7]. Limit values have been proposed and are suggested to be implemented in June 2025, with further reductions in limit values expected every fifth year ultimately leading to net-zero emissions by 2045 [7]. The Swedish National Board of Housing, Building and Planning also proposed a separate mandatory declaration of biogenic carbon storage in wood-based products from 2027 [7]. On a European level, the updated Bioeconomy Strategy [3] explicitly states the use and development of bio-based products to achieve circularity and sustainability in the EU. According to this strategy, a more robust and sustainable European bio-based sector could accelerate the substitution of non-renewable resources. This aligns with the EU’s commitments under the Paris Agreement [3]. Furthermore, the EU Taxonomy aims to clearly define what is considered sustainable when scaling up sustainable investments in the EU. Here, sustainably sourced bio-based and other raw materials are mentioned as contributing to the transition to a circular economy [9].

Energy use for space heating is a critical issue in building design in the Nordic countries. With the region’s cold and dark winters, there is a strong focus on the building envelope’s energy efficiency. In Europe, strict energy performance requirements have led to building designs with building envelopes with high thermal resistance [9]. For example, in southern Sweden, the maximum permitted average heat transfer coefficient (U-value) for a single-family house is 0.30 [W/(m^2^ K)]. Additionally, the maximum allowed total energy use for space heating and hot water combined is 40 kWh per m^2^ of floor area and year [10]. Therefore, sufficient thermal insulation is a crucial component in Swedish building design and is a significant part of the climate impact of a new building [11].

Bio-based insulation materials have relatively high sorption isotherms [12], especially in comparison to mineral wool and polystyrene-based insulation materials. The hygroscopicity of bio-based insulation materials influences their hygrothermal behaviour [13]. Therefore, it is a relevant material property to consider when studying the overall hygrothermal performance of a building, including its energy performance. Previous studies on the hygrothermal performance of bio-based insulation materials showed an overall high hygroscopicity [14]. However, when comparing similar bio-based insulation materials such as hemp fibre insulation, significant differences between individual products have been found [15,16]. 

This study assessed the hygrothermal properties of three bio-based insulation materials that have the potential to be locally sourced in Sweden. Thermal insulation batts of eelgrass, hay (roadside grass), and wood fibre were investigated. In addition, the hygrothermal properties of mineral wool (stone wool) insulation were studied as a reference material. The results can be used to make more accurate predictions of the energy performance of building envelopes with bio-based insulation materials. Accurate material properties are essential in numerical modelling because the accuracy and reliability of any calculation depends on the quality of the input data.

## 2. Materials and Methods

A combination of laboratory methods was used to evaluate some of the most essential hygrothermal properties of the insulation materials mentioned above. Material properties were measured on the material level and building component level. The following methods were used: dynamic vapour sorption (DVS), transient plane source (TPS), and sorption calorimetry. Moreover, moisture buffering of the insulation materials was measured using a climate chamber. In addition, the materials were tested on a building component level in a hot box [17] that exposed the materials to a steady-state climate, simulating in-use conditions, e.g., an external wall. Using this combination of laboratory measurements, the hygrothermal properties of the thermal insulation materials were investigated, compared, and analysed. 

### 2.1. Insulation Materials

Four different thermal insulation materials were investigated. These insulation materials were chosen for their local availability in Sweden, both the availability of their constituent raw materials and the availability of commercial products based on these raw materials. The bio-based materials were chosen to represent a diverse source of raw materials. This study focused on insulation batts suitable for thermal insulation of timber framed walls. With load-bearing wooden studs and an infill of thermal insulation material, such walls are the most common construction type for newly built single-family dwellings in Sweden (>98%). They are also increasingly common for multifamily houses [18,19].

**Figure 1:**
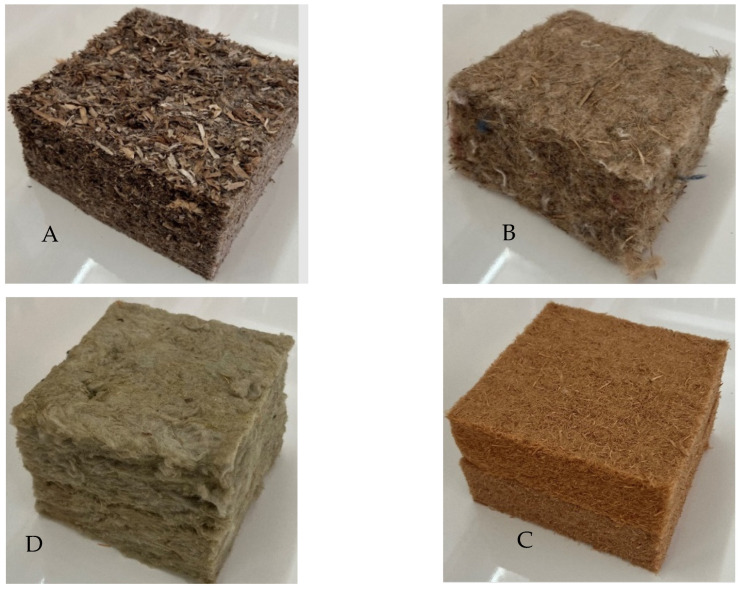
Samples of the insulation materials investigated in this paper. Clockwise, starting in the top left corner: eelgrass (**A**), grass (**B**), wood fibre (**C**), and stone wool (**D**).

The thermal insulation products investigated were based on one of the following raw materials: eelgrass, grass, wood fibre, and stone wool, see photographs in Figure 1. Material properties for the insulation materials, density, thermal conductivity, and specific heat capacity, can be found in Table 1. Note that eelgrass insulation is not (yet) a commercial thermal insulation product, but a similar product is being produced in Denmark and marketed as acoustic insulation. Therefore, the eelgrass-based thermal insulation material had no declared material properties. Instead, density and thermal conductivity were based on estimates based on the acoustic panel from the same manufacturer.

Eelgrass (*Zostera marina*) is a type of seagrass found along the Danish and Swedish coasts, constituting a vital carbon sink [24]. Even though eelgrass meadows in Swedish waters are scarce, there is an abundance of eelgrass along the coasts of southern Sweden and in Danish waters [25]. The eelgrass for insulation material is harvested when washed ashore after periods of intense waves, which are frequent in the area [26]. In contrast, grass insulation is based on “waste” grass from, amongst other sources, lawns and grass clippings from road verges, waterways, and motorways [27]. Wood fibre is a by-product of the Swedish forestry industry [28,29]. Examples of other bio-based insulation materials with the potential to be locally sourced but not included in the current study are hemp fibre, flax fibre, sheep’s wool, and cellulose-based insulation materials. All of these have the potential to be harvested, produced, and used locally in Sweden [30].

### 2.2. DVS—Dynamic Vapour Sorption

The DVS method is a commonly used technique for measuring the sorption isotherms of materials, which is the relationship between the equilibrium moisture content of the material and the relative humidity of its surrounding environment [31]. This method involves exposing a sample of the material to a controlled humidity environment and measuring the change in mass of the sample as it absorbs or desorbs moisture, see, e.g., [32].

Sorption isotherms were conducted at a temperature of 20 °C using automated sorption balances (DVS Advantage from Surface Measurement Systems Ltd., Alperton, UK). During these tests, the sample mass was measured with a resolution of 0.1 µg while the relative humidity (RH) was incrementally increased in pre-programmed steps: for wood and grass-based materials, 0%, 25%, 50%, 80%, 90% RH and, after that, desorption was conducted according to the same steps. The eelgrass sample was measured using the same regimen, except that the highest RH was 95%. Stone wool insulation was first exposed to desorption and then to absorption. A DVS instrument produces specific RH levels by combining dry and water-saturated nitrogen gas streams. Using the method outlined in [33], the generated RH’s accuracy (±1.5%) was verified. The allotted time to achieve equilibrium at each RH level was specified as a fixed (long) duration. Each experiment took ten days, with every step being at equilibrium [33,34].

The wood fibre insulation material contained ammonium phosphate (NH_4_)_3_PO_4_, which at 20 °C reaches an equilibrium relative humidity of 80% [35]. To capture this behaviour accurately, ten extra steps between 75 and 85% RH were added to the test of the wood fibre insulation sample.

### 2.3. Sorption Calorimetry

Sorption calorimetry is a technique used to measure the heat of sorption, which refers to the heat released or absorbed when a substance interacts with or adsorbs another substance, in this case, water vapour. Except for a study on wood [36], sorption calorimetry is here for the first time used to study the sorption energetics of construction materials. Previous studies using this method have been on pharmaceutical compounds and other biomolecules [37,38,39,40]. Sorption calorimetry provides insights into the energetics of the sorption processes (enthalpy of sorption/mixing) allowing for a better understanding of a material’s interaction with moisture. This understanding helps to determine whether excess heat from the interaction between water and material will influence the overall thermal performance of building assemblies. In the sorption calorimeter used, continuous measurements of the thermal power of vaporisation from a water source and the thermal power of sorption in a sample are made. Considering both as positive for exothermal processes, the thermal power of sorption is positive, while the thermal power of vaporisation is negative. These two thermal powers allow the continuous evaluation of changes in a triplet of information throughout the measurements: sample relative humidity, moisture content, and mixing enthalpy. 

Water that is bound to a material has other properties than bulk water. This is, for example, seen in the fact that more heat is needed to dry a material than is needed to vaporise the corresponding amount of bulk water. This excess heat by which water is bound to materials is most conveniently quantified as the heat release when the state of water changes from (bulk) liquid to bound, this is termed the mixing enthalpy (J/g_water_). It is a function of the moisture content as the interactions between water and material are much more intense at lower relative humidities [36]. However, because of hysteresis effects, measuring the mixing enthalpy directly is impossible. Therefore, the interaction between water vapour and material (the sorption enthalpy) is studied in the sorption calorimeter. The mixing enthalpy is then calculated by subtracting the constant condensation enthalpy. More information on the sorption calorimetric method is found in [36].

The sorption calorimeter utilised in this study is a novel, as yet unpublished instrument designed for investigating solid–vapour interactions. This instrument employs two isothermal heat conduction calorimeters, forming a double twin calorimeter that simultaneously and independently measures the heat production rate (thermal power) during both the vaporisation of water from a vapour source and the absorption of the same vapour in a sample. Throughout the measurement process, water vaporised at the water source diffuses down a tube to the sample, increasing its moisture content and relative humidity. More information about the type of sorption calorimeter used is found in [37,40].

The samples for sorption calorimetry were dried for 24 h at 60 °C under vacuum before testing. Dry masses of the tested samples ranged from 20 to 31 mg, and the measurements were conducted at 25 °C. The calorimeter was calibrated using electrical heaters, and the maximal output signal (approximately 800 μW) was measured with drying agents (molecular sieves).

### 2.4. MBV—Moisture Buffer Value

Moisture buffer value (MBV) indicates the ability of hygroscopic materials to regulate humidity levels. High moisture buffering demonstrates that a material can effectively absorb and release moisture. Which, if open to diffusion from the indoor environment, can help to prevent problems such as condensation, mould growth, and indoor air quality issues. It can also help reduce ventilation needs, leading to lower energy needs [41]. Therefore, surface materials with high moisture buffering can be beneficial for improving the indoor environment in buildings. A material sample is typically subjected to steady temperature conditions and fluctuating relative humidity to investigate the moisture buffering of building materials. The weight change (change in mean moisture content) is then measured over time. A standardised test method to determine moisture buffering value is the NORDTEST method [42], which involves exposing a material sample to cyclic changes (24 h) in temperature and humidity and measuring the moisture uptake and release rate. In this study, an adaptation of the NORDTEST protocol was used to determine moisture buffering properties of the insulation materials. A climate test cabinet (CTS C-20/1000) exposed the insulation materials to a regimen of 8 h at 75% RH, followed by 16 h at 33% RH. Between these two RH levels, there was a transition period of 30 min to allow the climate cabinet to adjust to the new relative humidity level. Therefore, the regimen consisted of 8 h at 75% RH, followed by 30 min of transition in which the climate cabinet adjusted towards 33% RH. Then, 16 h at 33% RH was followed by 30 min, during which the climate cabinet adjusted towards 75% RH. This gave a total interval of 25 h, compared to an interval of 24 h suggested in the NORDTEST method. The temperature inside the climate cabinet was kept stable at 20.5 ± 1.0 °C. Three balances (OHAUS Scout) were placed inside the test cabinet. Material samples were suspended underneath these balances and measured continuously. Three samples were tested for each material. 

Before the material samples were exposed to the moisture buffer regimen, they were dried for 24 h at 40 °C, 24 h at 50 °C, and then dried at 60 °C until they reached equilibrium. Equilibrium was considered reached when there was a variation in weight of less than 0.01% between three consecutive weighings at 24 h intervals. Once equilibrium was reached, the dried samples were placed in a climate chamber with 60 ± 5% relative humidity and 20 ± 0.5 °C. The samples were in the climate chamber until they reached equilibrium, defined as a variation in weight of less than 0.01% between 3 consecutive weighings at 24 h intervals. The samples were clad in aluminium tape on five sides, leaving one surface of approx. 150 × 150 mm^2^ open with the exact surface area measured for each sample. 

Each balance was placed inside a closed plastic container with a desiccant to keep the relative humidity inside the container low. A hole was made in the bottom of the plastic container to allow for below-balance weighing. Air flows can influence the moisture buffer capacity of materials. Air velocity inside the test cabinet was measured and found to be 0.14 ± 0.10 m/s, somewhat higher than the interval recommended by the NORDTEST protocol (0.10 ± 0.05 m/s) [43]. 

### 2.5. TPS—Transient Plane Source

The transient plane source (TPS) method is a non-destructive, non-invasive, and quick method to measure the thermal properties of various substances, including liquids, solids, and powders [44]. TPS has previously been used for bio-based insulation materials [44,45,46,47,48]. The TPS method involves a probe with a combined heater and temperature sensor. This probe is usually placed between two layers of the tested material. A controlled current is passed through the probe to generate a constant thermal power, which causes it to heat up. The temperature of the heating element is then measured over time, and the material’s thermal conductivity and thermal diffusivity can be calculated based on the rate of temperature change. 

In this study, the hardware used was a HotDisk TPS 2500 S (HotDisk AB, Göteborg, Sweden) with a testing probe with a radius of 29.52 mm (Kapton 5599); see [49]. Samples were conditioned by drying in a vacuum oven for a week at 60 °C. Then, the samples were placed in a ‘dry box’ where the relative humidity was kept at 10 ± 5% and the temperature was at 21 ± 0.5 °C during testing. The samples were placed in the ‘dry box’ immediately after drying and were allowed to acclimate before testing. One test consisted of a repetition of three measurements; after one test, the probe was moved, and another was performed until three tests (of three repetitions) were carried out. A test consisted of one measurement period of between 320 and 640 s, depending on the material, followed by a waiting period 36 times as long as the measurement period to allow the material to cool down. The applied heat varied from 27 mW to 87 mW, causing temperature increases between 2.8 K and 4.5 K.

### 2.6. Hot Box

The hot-box method is an experimental test used to measure the thermal performance of building components, such as walls, roofs, and windows. A hot-box test setup consists of two boxes or chambers with controlled temperature conditions. The test specimen (i.e., the building component) is placed between the hot and cold sides of the boxes. Then, the interior and exterior sides of the hot box are maintained at different temperatures, which creates a temperature gradient over the test specimen. This temperature gradient causes heat to flow through the specimen, and the heat transfer rate is measured and analysed to determine the thermal performance of the specimen. The hot-box method can assess several essential properties of building components, including thermal conductivity, thermal resistance, and overall thermal transmittance (U-value). 

This study used a custom-made version of the hot-box equipment, explained in detail in [17]. This version of the hot box is similar to, e.g., [50,51,52], but has a greater possibility to measure and control relative humidity. This version of the hot box was primarily built for comparative analysis and not absolute numbers. However, the acquired U-values have been benchmarked with other testing methods with good results, especially for non-hygroscopic insulation materials [17]. The hot-box setup in this study is what the hot-box standard calls a ‘calibrated HotBox’ [53], where heat leakage between the hot box and the surrounding laboratory was considered by calibrating the setup. Calibration was performed by increasing the temperature in the climate cabinet and the hot box to 30 °C, thereby assuming no heat transfer through the specimen. This meant all energy used for heating is leakage, and the heat-loss coefficient of the test setup is obtained. 

Specimens for the hot-box tests were created by glueing two insulation batts together. The specimen size was 1 × 1 m^2^, and they were mounted to an 85 mm PIR frame within the hot box, meaning that the interior surface area was 0.83 × 0.83 m^2^, which is the assumed area used in the U-value calculations. All tested insulation materials were fixed to a timber frame for structural stability. All insulation specimens were covered with an external wind barrier (5000 s/m) to simulate a more realistic setup while reducing the impact of air leakage through the specimens, which would not occur in a wall. 

**Figure 2:**
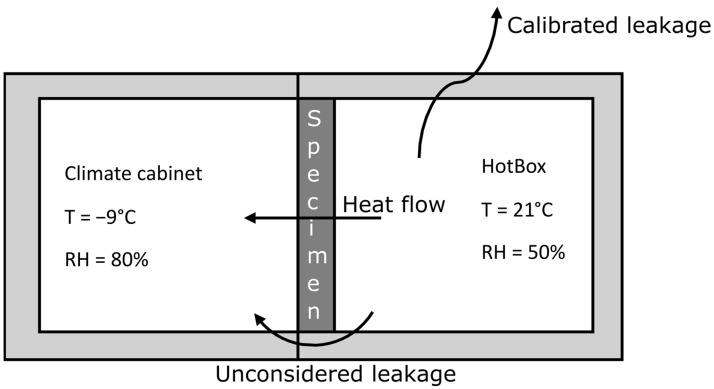
The principal figure of the hot-box test setup shows the test conditions and general assumptions. The specimen, which consisted of insulation and an exterior wind barrier, was placed between the hot and cold sides. Throughout the test, a temperature difference of 30 K was maintained.

The steady-state condition in the hot box was an indoor temperature of 21 °C and an outdoor temperature of −9 °C, creating a temperature difference of 30 K over the specimen. After placing the specimens in the hot box, they were allowed to reach a steady state within two days before testing. Specimens were then exposed to a measurement period of five days. Temperature and humidity were measured with Vaisala HMP110 (with an accuracy of ±0.2 K and 1.5% relative humidity) sensors calibrated before the test. The sensors were placed on both the ‘hot’ and ‘cold’ sides, as well as on the specimen surface. The temperature measurements used as ΔT were the mean temperature of the two sensors placed centrally in the hot box and the mean value of the two placed centrally in the climate cabinet, see Figure 2 for a principal figure. Heating the hot box with an electric heater and assuming all electricity becomes heat, energy for heating is acquired. Temperature and electricity were measured and logged every minute; however, the action of the PID controller was not that rapid. Therefore, aggregated hourly data were used.

## 3. Results and Discussion

The results from the experiments, followed by a short discussion, are presented here per measurement method. 

### 3.1. DVS—Dynamic Vapour Sorption

**Figure 3:**
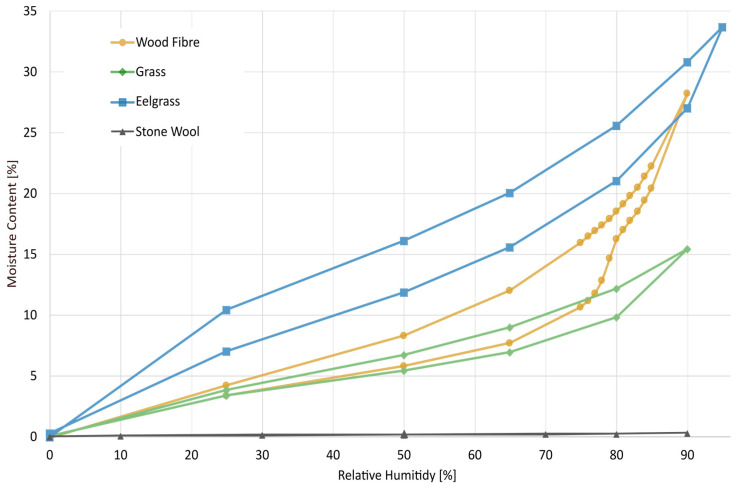
Sorption isotherms obtained from the DVS measurements for the four materials studied. The lower curve represents absorption, while the upper curve depicts desorption. Data points mark equilibrium measurement points. Desorption was initiated at 90% RH for all samples, except for the eelgrass sample, which was started at 95% RH. Additional data points were included for the wood fibre insulation to capture the effect of the added salt.

The results from the DVS test can be seen in Figure 3, and the most noticeable result is how much more hygroscopic the tested bio-based insulation materials are than the stone wool. Furthermore, the results show how different bio-based insulation materials have significant differences in sorption isotherms. The tested wood fibre insulation largely follows a spruce sorption isotherm [54], but the effect of the fire retardant salt on the isotherm is noticeable. The clear increase in the wood fibre sorption curve at 75–80% RH correlates well with the ammonium phosphate ((NH_3_)_2_HPO_4_) content in the material, as stated by the manufacturer. Interestingly, this increase in the absorption isotherm could not be seen as a corresponding decrease in the desorption. Furthermore, there is a significant difference in hysteresis between the bio-based materials, whereas eelgrass has a hysteresis with almost a 5% difference in moisture content throughout the hygroscopic range. In contrast, the grass insulation had much lower hysteresis, especially at lower relative humidity, and the wood fibre had an increasing hysteresis with increasing RH.

By switching from absorption to desorption at 90% (or 95% for the eelgrass), the presented desorption curve is not an actual desorption curve at high RHs [32]. Instead, the presented sorption curves are scanning curves, at least at high relative humidity. However, insulation materials should never be subjected to such high moisture states so that they experience true desorption in real-world applications. The reason for measuring the eelgrass sample up to 95% RH was to see if the sea salt was visible in the isotherm, which it was not. For all sorption tests, the choice of equilibrium criterion is essential and influences the results [34]. However, for the wood fibre and grass insulations, the results from this study show similarity to the sorption curves from the literature [55,56].

### 3.2. Sorption Calorimetry

**Figure 4:**
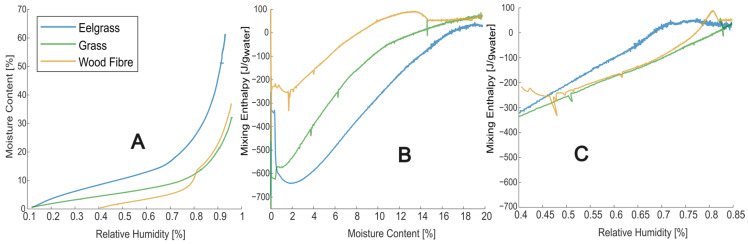
Results from the sorption calorimetry experiments. (**A**) Sorption isotherm from the sorption calorimetry is similar to the sorption isotherm from the DVS but faster. The wood fibre sample is likely not in equilibrium at lower moisture contents. (**B**) Enthalpy of mixing as a function of the moisture content, negative enthalpy means an exothermic reaction. (**C**) Enthalpy of mixing as a function of relative humidity in the range of interest in building applications. Note that the wood fibre measurement is not made at equilibrium at lower RHs, but the result still shows the endothermal effect of the added salt at high RHs.

In Figure 4A, the sorption curves from the sorption calorimetry are presented. It is seen that the wood fibre sample could not be measured close to equilibrium conditions at the start of the measurement (at lower RHs), as it was slow in absorbing moisture. However, at higher RHs, the values correspond well with those measured by DVS as the rate of moisture uptake by the sample decreases throughout the measurement, and even if it is a non-equilibrium condition at the start, it can come closer and closer to such conditions during measurement. Note that the samples had been dried before the measurement, and the wood fibre insulation measurement was repeated several times with different samples and similar results. For eelgrass and grass insulation, the results correspond well with the sorption measurements by DVS; compare Figure 3 to Figure 4A. Figure 4B shows the heat of mixing as a function of the moisture content, negative heat meaning an exothermal process. All tested materials show a lower absolute heat of mixing than pure wood samples [36], which is surprising, especially for the wood fibre insulation sample. Additionally, the influence of the added salt in the wood fibre sample is very noticeable as an endothermic ‘hump’ on the curve, see Figure 4B. Figure 4C presents the heat of mixing as a function of relative humidity, though it is not physically accurate, it is a more intuitive picture of how the materials behave in the hygroscopic range. For instance, notice the difference between the salt ‘hump’ in the wood fibre sample between Figure 4B,C. No sorption calorimetry was conducted on the stone wool, as it absorbs too little moisture to give reliable results with this method.

Relative humidity, moisture content, and mixing enthalpy are determined over time. Ideally, a sample readily absorbs the moisture that reaches it, ensuring that the measurement is made close to sorption equilibrium throughout the measurement. The sorption calorimetric method does not work well with samples with ‘delayed sorption’. This is usually not a problem for finely divided materials (like the materials in this study). Still, the wood fibre insulation gave odd results, indicating a delayed sorption. In the results, the sorption isotherm for the wood fibre insulation does not start at low RHs but at around 40%. However, these results are still included as the sorption calorimeter clearly shows the increased water vapour sorption of salt added to the wood fibre insulation.

The sorption rate cannot be controlled except by adjusting the sample size, which was at its maximum for these low-density samples. Larger sample holders should be manufactured for future sorption calorimetry tests on low-density materials. The sorption of the wood fibre material was not fast enough to maintain equilibrium, but the measurement worked well for the other two bio-based materials. It is unknown why the wood fibre material had slower sorption, but this may be related to the so-called non-Fickian behaviour seen in many wood studies [57,58]. Note that the non-equilibrium problem only affects the relative humidity measurement, mainly at low humidity levels when the diffusion rate is at its highest, as it is driven by the difference in relative humidity between the water source and the sample. The moisture content and mixing enthalpy remain robust and measured accurately regardless of equilibrium status. The moisture content is calculated from the integral of the thermal power of vaporisation from the water source. The mixing enthalpy is derived from the ratio of vaporisation thermal powers, both of which are independent of equilibrium.

### 3.3. MBV—Moisture Buffer Value 

**Figure 5:**
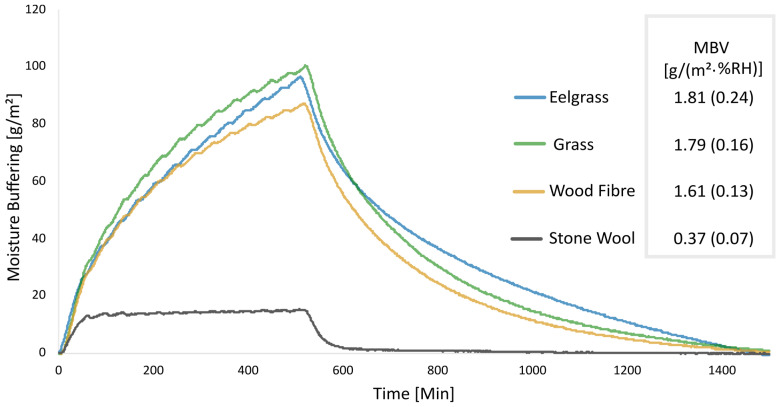
To the right are the moisture buffer values [g/(m^2^·%RH)] for each of the four materials with standard deviation in parentheses, an average of three samples for two absorption/desorption cycles of 8 h/75%RH and 16 h/33% RH. The curve shows the raw data from one measurement per material, for one balance. The test also includes a 30 min transition period between high and low humidity.

Figure 5 shows the results of the moisture buffer value tests as mean values for three samples per material, with corresponding standard deviations. The MBV of the bio-based materials can be classified as “Good”, while the stone wool can be classified as “Limited” according to the NORDTEST protocol [59]. Additionally, Figure 5 shows the raw data from one measurement cycle of 25 h (adsorption and desorption) for the four different materials. This shows how the materials behave when being exposed to cycles of higher and lower relative humidity. Similar to the findings in the DVS tests, it is clear how much more hygroscopic the bio-based materials are compared to stone wool. Interestingly, in the MBV tests, the difference between the bio-based materials is not as significant as in the sorption experiments. This shows that the moisture buffer value is similar for the bio-based insulation samples.

Comparing the DVS and MBV measurements highlights that relying solely on sorption curves might not accurately reflect a material’s moisture buffer capacity. While significant discrepancies emerge among hygroscopic materials in DVS assessments, these differences are absent in MBV evaluations. Despite similar density and porosity across the tested materials, variations in vapour permeability likely account for these contrasting results. This variance could stem from nuanced differences in vapour permeability among the materials. Even though the balances were placed inside plastic containers with a desiccant, it was found that they reacted to changes in relative humidity inside the climate cabinet. Measurement files were therefore calibrated as explained in [60]. 

### 3.4. TPS—Transient Plane Source

**Figure 6:**
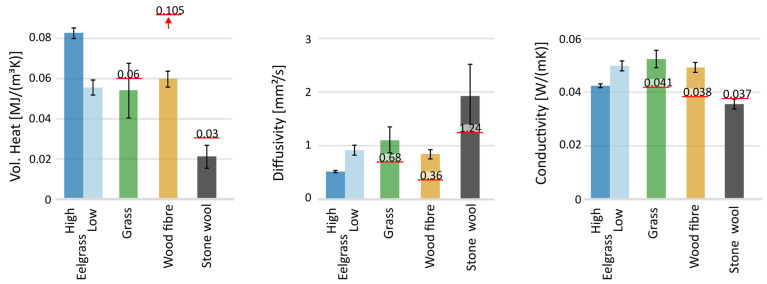
Results from the TPS measurements. The red line is the manufacturer’s declared value. Whiskers indicate standard deviation. ‘High’ and ‘Low’ for the eelgrass indicate which side was measured. To the left is the volumetric heat capacity, in the middle is the thermal diffusivity, and to the right is the thermal conductivity.

Figure 6 shows the experimental results and the standard deviation from the TPS test. For the eelgrass insulation, there was an apparent difference in density between the sides, likely from the manufacturing process where heat was applied on one side in the press. Therefore, for clarity and the possibility of distinguishing them, the two sides are split and presented separately as “high” and “low” density. 

Looking at the measured volumetric heat capacity, it is clear that stone wool reacts very differently to the bio-based materials. There was a difference between the two sides of the eelgrass insulation as well. The variation in results was most pronounced for the grass insulation, likely due to its somewhat uneven structure and diverse composition of fibres. Additionally, the measured volumetric heat capacity was consistently lower for all materials when compared to the data on density and specific heat capacity provided by the manufacturers. For the thermal diffusivity, the stone wool insulation had a noticeably higher value and a larger spread than the other materials. Moreover, the measured thermal diffusivity was significantly higher than expected, given manufacturers’ data. The experimental result for the thermal conductivity shows that all bio-based materials have a higher value than stone wool insulation, which is to be expected [61,62]. Additionally, the bio-based materials have the most significant difference compared to the declared values. Furthermore, both the grass and wood fibre insulation showed higher thermal conductivity than declared. While the opposite was found for the stone wool.

The TPS experiments were conducted in a ‘dry box’ to maintain stable conditions, but conditions still varied slightly. A drying agent was placed in the box with the sample, leading to a systematically higher relative humidity in the last measurement series than in the first. However, no systematic trends in the results could be seen due to this. The comparatively large spread in results is likely due to the nature of the very porous insulation materials. This was slightly compensated for with the choice of a large sensor. However, a large sensor has a more significant variation in the thermal contact between the sensor and samples. All bio-based materials showed a higher thermal conductivity in the TPS tests than the values declared by the manufacturers using steady-state measurements, likely completed with a hot-plate method. The differences are even more significant than seen in [44] due to the low conductivity of the tested insulation materials and likely due to the hygroscopic nature of the materials. A temperature shift naturally changes the relative humidity and induces moisture transport, which makes a difference between transient and steady-state measurements (e.g., Hot-plate). 

### 3.5. Hot Box

**Figure 7:**
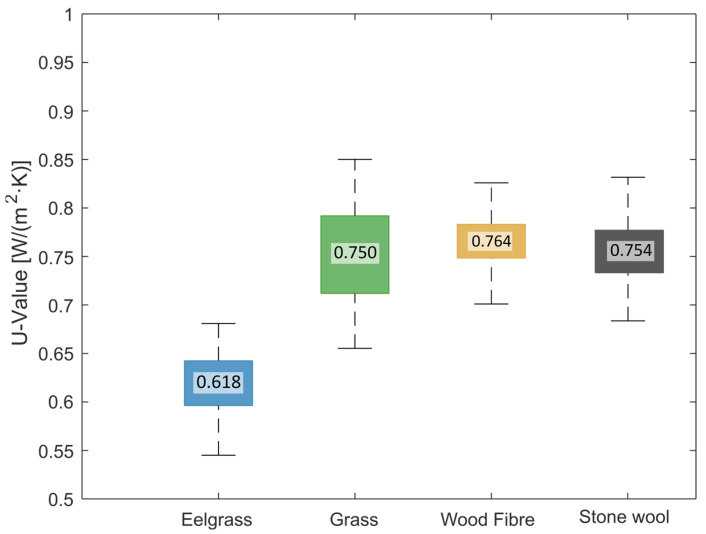
Thermal transmittance was measured in the hot box and presented as boxplots of the U-value for a 1 m^2^ specimen. The number in the middle is the mean value; whiskers show the spread, and the box indicates a 25% and 75% confidence interval. Note that the materials have different thicknesses and should not be directly compared.

Results from the hot-box tests are shown as a boxplot in Figure 7 as values for a 1 m^2^ specimen. It is important to note that the thickness is not the same for the specimens (see Table 1). Thus, the U-value should not be compared directly between materials. The U-value range varies significantly across insulation types, with wood fibre insulation having the lowest range and eelgrass insulation showing surprisingly low thermal transmittance when tested with the denser side inwards. The wood fibre and stone wool specimens show similarities with previous research [17].

Note that the compared material thicknesses, as seen in Table 1, ranged from 45 mm for wood fibre and stone wool to 60 mm for eelgrass and 80 mm for grass. The calibration test was carried out with the stone wool insulation, and with the grass specimen being almost twice as thick, the leakage and the resulting U-value are likely overestimated. The same or similar thicknesses would have reduced this uncertainty. By having an exterior weather barrier on all specimens, the influence of the material’s vapour resistance has a negligible effect. 

## 4. Analysis

**Figure 8:**
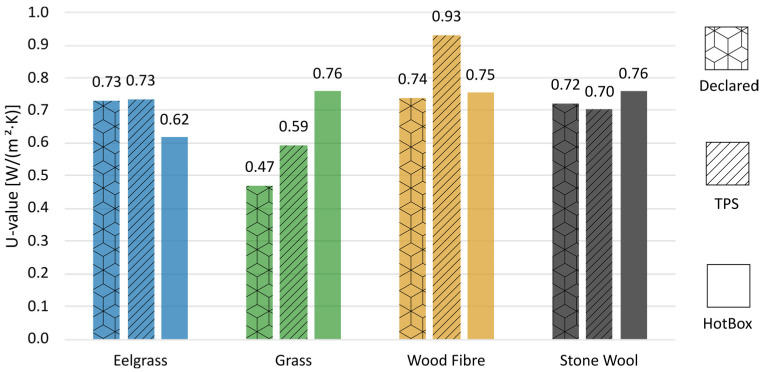
Comparison of thermal transmittance (U-Value) from the expected U-value from the declared thermal conductivity, measured thermal conductivity, and measured U-value from the hot box test.

Figure 8 illustrates a comparative analysis between the thermal conductivity values declared by manufacturers and the outcomes from both TPS and hot box experiments. The standard approach of calculating expected U-values from thermal conductivity, as outlined in [63], was employed for this comparison. Notably, while the TPS test accurately evaluates the insulating capacities of the non-hygroscopic stone wool, the same level of accuracy is not observed for the more hygroscopic bio-based insulation materials.

A substantial variance emerges between the thermal conductivity values and the measured U-values. In the hot box test, for instance, the grass insulation exhibited a higher U-value than that derived from the TPS test. Conversely, both eelgrass and wood fibre were found to offer better insulation performance compared to what their thermal conductivity values alone would suggest. This phenomenon aligns with findings from prior research, particularly concerning wood fibre insulation [17].

The underlying reasons for the disparities observed among the bio-based insulation materials remain unclear. However, notable distinctions exist between them, such as eelgrass exhibiting the highest sorption capacity (as depicted in Figure 3) along with the greatest density (refer to Table 1), while grass demonstrates the opposite trend. This observation prompts speculation that the latent heat of the condensation might be a contributing factor to these differences, a possibility supported by numerical analyses in previous studies [64]. Further research is warranted to explain the precise mechanisms driving these discrepancies and their implications for the thermal performance of bio-based insulation materials.

## 5. Conclusions

The sorption properties of bio-based insulation materials not only distinguish them significantly from stone wool but also reveal notable variations among themselves. Despite these differences, all bio-based materials exhibited similar moisture buffer values despite having distinct sorption isotherms. This suggests that the sorption rate holds greater importance than isotherms, implying potential disparities in vapour resistance among the materials. Interestingly, these findings diverge from the results of sorption calorimetry tests, where wood fibre displayed a notably lower sorption rate at lower relative humidity compared to other bio-based materials. However, all bio-based materials exhibited similar mixing enthalpy, with each demonstrating lower values than pure spruce samples [36]. It is worth noting that this study marks the first investigation of sorption calorimetry conducted on insulation materials, providing novel insights into their hygrothermal behaviour.

Analysis of thermal performance revealed significant discrepancies between the measured values and manufacturers’ declarations. In TPS tests, all of the bio-based materials exhibited higher thermal conductivity than the manufacturers declared. Additionally, hot box experiments underscored a substantial difference between the actual insulating capacity of bio-based insulations and predictions derived from their thermal conductivity. This highlights the inadequacy of relying solely on thermal conductivity measurements for estimating the thermal performance of hygroscopic insulations in building contexts. Conversely, for the non-hygroscopic stone wool insulation, calculating U-values from thermal conductivity proved to be an accurate method for assessing thermal performance.

This study comprehensively measured seven key hygrothermal properties—sorption isotherms, thermal conductivity, thermal diffusivity, volumetric heat capacity, mixing enthalpy, moisture buffer value, and U-value—of three novel bio-based insulation materials alongside conventional mineral wool. These results provide valuable input for numerical simulations aimed at evaluating the hygrothermal performance of buildings utilising bio-based insulation materials.

The study’s scope focused on investigating locally sourced bio-based insulation materials in Sweden, specifically eelgrass, grass, and wood fibre. However, given the global availability of these raw materials, the results likely hold relevance beyond Sweden and can be generalised for materials from similar sources worldwide. This broad applicability underscores the potential of bio-based insulation materials to contribute to sustainable building practices on a global scale.

## Figures and Tables

**Table 1:** Material properties as declared by the material manufacturers.

Insulation Material	Density, ρ [kg/m^3^]	Thermal Conductivity, λ [W/(mK)]	Specific Heat Capacity, c [J/(kgK)]	Thickness [mm]	Ref.
Eelgrass	120 *	0.05 *	no data available	60	[20]
Grass	40	0.041	1500	80	[21]
Wood fibre	50	0.038	2100	45	[22]
Stone wool	29	0.037	1030	45	[23]

* Data from a similar product from the same manufacturer.

## Data Availability

The raw data supporting the conclusions of this article will be made available by the authors on request.
